# Crystal structure of 5-chloro-2-(2-fluoro­phen­yl)-3-methyl­sulfinyl-1-benzo­furan

**DOI:** 10.1107/S2056989015013948

**Published:** 2015-07-31

**Authors:** Hong Dae Choi, Uk Lee

**Affiliations:** a Department of Chemistry, Dongeui University, San 24 Kaya-dong, Busanjin-gu, Busan 614-714, Republic of Korea; b Department of Chemistry, Pukyong National University, 599-1 Daeyeon 3-dong, Nam-gu, Busan 608-737, Republic of Korea

**Keywords:** crystal structure, benzo­furan, 2-fluoro­phen­yl, C—H⋯O hydrogen bonds, F⋯π and S⋯F contacts

## Abstract

In the title compound, C_15_H_10_ClFO_2_S, the dihedral angle between the mean planes of the benzo­furan ring [r.m.s. deviation = 0.007 (1) Å] and the 2-fluoro­phenyl ring is 32.53 (5)°. In the crystal, mol­ecules related by inversion are paired into dimers *via* two different C—H⋯O hydrogen bonds. Further, Cl⋯O halogen bonds [3.114 (1) Å], and F⋯π [F-to-furan-centroid distance = 3.109 (1) Å] and S⋯F [3.1984 (9) Å] inter­actions link these into a three-dimensional network.

## Related literature   

For the pharmacological properties of benzo­furan compounds, see: Aslam *et al.* (2009 ▸); Galal *et al.* (2009 ▸); Howlett *et al.* (1999 ▸); Wahab Khan *et al.* (2005 ▸); Ono *et al.* (2002 ▸). For a related structure, see: Choi & Lee (2014 ▸). For further synthetic details, see: Choi *et al.* (1999 ▸). For a review of halogen bonding, see: Politzer *et al.* (2007 ▸).
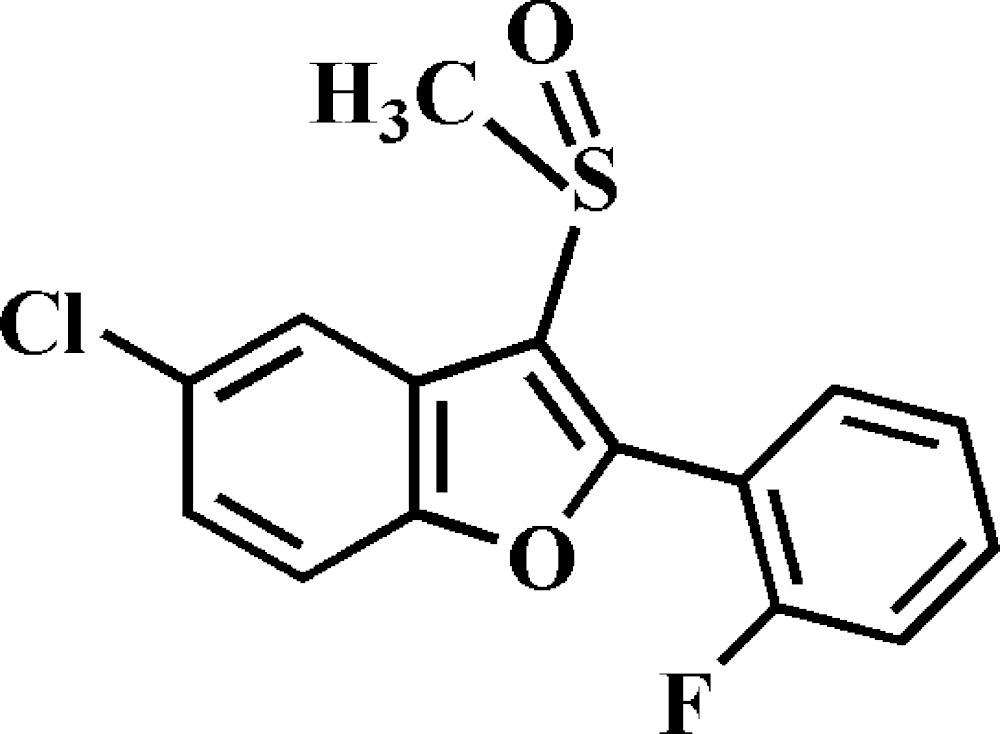


## Experimental   

### Crystal data  

C_15_H_10_ClFO_2_S
*M* *_r_* = 308.74
Triclinic, 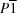

*a* = 7.9626 (1) Å
*b* = 8.3518 (1) Å
*c* = 10.7127 (2) Å
α = 92.758 (1)°
β = 95.509 (1)°
γ = 112.373 (1)°
*V* = 652.97 (2) Å^3^
*Z* = 2
Mo *K*α radiation
μ = 0.46 mm^−1^
*T* = 173 K
0.45 × 0.36 × 0.32 mm

### Data collection  

Bruker SMART APEXII CCD diffractometer
Absorption correction: multi-scan (*SADABS*; Bruker, 2009 ▸) *T*_min_ = 0.690, *T*_max_ = 0.746
12250 measured reflections
3263 independent reflections
3030 reflections with *I* > 2σ(*I*)
*R*_int_ = 0.023

### Refinement  

*R*[*F* ^2^ > 2σ(*F* ^2^)] = 0.030
*wR*(*F* ^2^) = 0.081
*S* = 1.05
3263 reflections
183 parameters
H-atom parameters constrained
Δρ_max_ = 0.34 e Å^−3^
Δρ_min_ = −0.26 e Å^−3^

### 

Data collection: *APEX2* (Bruker, 2009 ▸); cell refinement: *SAINT* (Bruker, 2009 ▸); data reduction: *SAINT*; program(s) used to solve structure: *SHELXS97* (Sheldrick, 2008 ▸); program(s) used to refine structure: *SHELXL2014* (Sheldrick, 2015 ▸); molecular graphics: *ORTEP-3 for Windows* (Farrugia, 2012 ▸) and *DIAMOND* (Brandenburg, 1998 ▸); software used to prepare material for publication: *SHELXL2014*.

## Supplementary Material

Crystal structure: contains datablock(s) I. DOI: 10.1107/S2056989015013948/fy2120sup1.cif

Structure factors: contains datablock(s) I. DOI: 10.1107/S2056989015013948/fy2120Isup2.hkl

Supporting information file. DOI: 10.1107/S2056989015013948/fy2120Isup3.cml

**Fig. 1.**
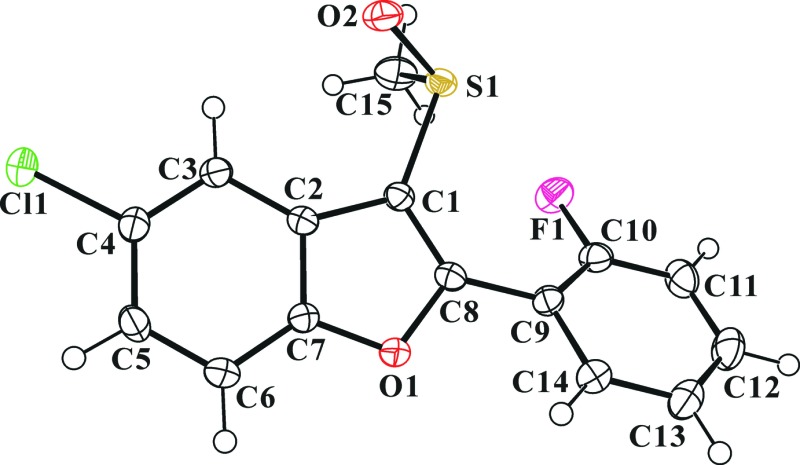
The molecular structure of the title compound with the atom numbering scheme. Displacement ellipsoids are drawn at the 50% probability level. H atoms are presented as small spheres of arbitrary radius.

**Fig. 2.**
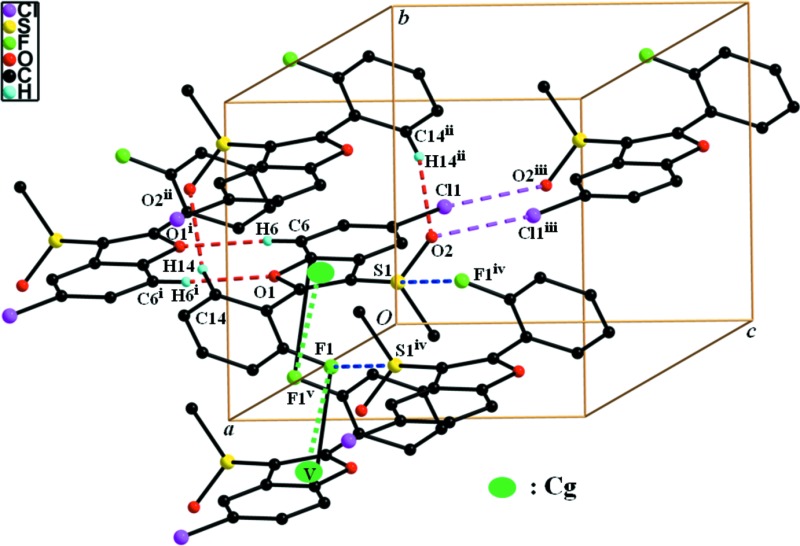
A view of the C–H···O, Cl···O, F···π and S···F interactions (dotted lines) in the crystal structure of the title compound. H atoms not participating in hydrogen-bonding were omitted for clarity. [Symmetry codes: (i) - x + 2, - y + 1, - z; (ii) - x + 1, - y + 1, - z; (iii) - x + 1 , - y + 1, - z + 1; (iv) - x, - y, - z ; (v) - x + 1, - y, - z.]

. DOI: 10.1107/S2056989015013948/fy2120fig1.tif
The mol­ecular structure of the title compound with the atom numbering scheme. Displacement ellipsoids are drawn at the 50% probability level. H atoms are presented as small spheres of arbitrary radius.

x y z x y z x y z x y z x y z . DOI: 10.1107/S2056989015013948/fy2120fig2.tif
A view of the C–H⋯O, Cl⋯O, F⋯π and S⋯F inter­actions (dotted lines) in the crystal structure of the title compound. H atoms not participating in hydrogen-bonding were omitted for clarity. [Symmetry codes: (i) − *x* + 2, − *y* + 1, − *z*; (ii) − *x* + 1, − *y* + 1, − *z*; (iii) − *x* + 1, − *y* + 1, − *z* + 1; (iv) − *x*, − *y*, − *z*; (v) − *x* + 1, − *y*, − *z*.]

CCDC reference: 1414408

Additional supporting information:  crystallographic information; 3D view; checkCIF report

## Figures and Tables

**Table 1:** Hydrogen-bond geometry (, )

*D*H*A*	*D*H	H*A*	*D* *A*	*D*H*A*
C6H6O1^i^	0.95	2.53	3.4756(16)	176
C14H14O2^ii^	0.95	2.44	3.3591(17)	163

Symmetry codes: (i) 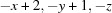; (ii) 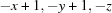.

## supplementary crystallographic information

### S1. Comment

Many compounds involving a benzofuran ring show significant pharmacological properties, such as antibacterial, antifungal, antitumor, antiviral and antimicrobial activities (Aslam *et al.* 2009; Galal *et al.*, 2009; Wahab Khan *et al.*, 2005), and are potential inhibitors of β-amyloid aggregation (Howlett *et al.*, 1999; Ono *et al.*, 2002). As a part of our continuing project on benzofuran derivatives (Choi & Lee, 2014), we report herein the crystal structure of the title compound.

In the title molecule (Fig. 1), the benzofuran unit is essentially planar, with a mean deviation of 0.007 (1) Å from the least-squares plane defined by the nine constituent atoms. The 2-fluorophenyl ring is essentially planar, with a mean deviation of 0.007 (1) Å from the least-squares plane defined by the six constituent atoms. The dihedral angle formed by the benzofuran ring and the 2-fluorophenyl ring is 32.53 (5)°. In the crystal structure (Fig. 2), molecules related by inversion are paired into dimers *via* two different C–H···O hydrogen bonds (Table 1), and a Cl···O halogen bond between the chlorine and the oxygen of the S═O unit [Cl1···O2^iii^ = 3.114 (1) Å, C4–Cl1···O2^iii^ = 171.16 (5)°] (Politzer *et al.*, 2007), F1···Cg^v^ [3.109 (1) Å] (Cg is the centroid of the C1/C2/C7/O1/C8 furan ring) and S1···F1^iv^ [3.1984 (9) Å] interactions, forming a three–dimensional network. [Symmetry codes: (i) - *x* + 2, - *y* + 1, - *z*; (ii) - *x* + 1, - *y* + 1, - *z*; (iii) - *x* + 1 , - *y* + 1, - *z* + 1; (iv) - *x*, - *y*, - *z* ; (v) - *x* + 1, - *y*, - *z*.]

### S2. Experimental

The starting material 5-chloro-2-(2-fluorophenyl)-3-methylsulfanyl-1-benzofuran was prepared by a literature method (Choi *et al.*, 1999). 3-Chloroperoxybenzoic acid (77%, 224 mg, 1.0 mmol) was added in small portions to a stirred solution of 5-chloro-2-(2-fluorophenyl)-3-methylsulfanyl-1-benzofuran (263 mg, 0.9 mmol) in dichloromethane (25 ml) at 273 K. After being stirred at room temperature for 8h, the mixture was washed with saturated sodium bicarbonate solution (2 × 10 ml) and the organic layer was separated, dried over magnesium sulfate, filtered and concentrated at reduced pressure. The residue was purified by column chromatography (hexane-ethyl acetate, 1:2 *v*/*v*) to afford the title compound as a colorless solid [yield 68% (188 mg); m.p. 431-432 K; *R*_f_ = 0.55 (hexane-ethyl acetate, 1:2 *v*/*v*)]. Single crystals suitable for X-ray diffraction were prepared by slow evaporation of a solution of the title compound (18 mg) in ethyl acetate (20 ml) at room temperature.

### S3. Refinement

All H atoms were positioned geometrically and refined using a riding model, with C–H = 0.95 Å for aryl and 0.98 Å for methyl H atoms and *U*_iso_ (H) = 1.2*U*_eq_ (C) for aryl and 1.5*U*_eq_ (C) for methyl H atoms. The positions of methyl hydrogens were optimized using the *SHELXL-2014/7* command AFIX 137 (Sheldrick, 2015).

### Crystal data

**Table:**

C_15_H_10_ClFO_2_S	*Z* = 2
*M**_r_* = 308.74	*F*(000) = 316
Triclinic, *P*1	*D*_x_ = 1.570 Mg m^−^^3^
*a* = 7.9626 (1) Å	Mo *K*α radiation, λ = 0.71073 Å
*b* = 8.3518 (1) Å	Cell parameters from 6796 reflections
*c* = 10.7127 (2) Å	θ = 2.7–28.4°
α = 92.758 (1)°	µ = 0.46 mm^−^^1^
β = 95.509 (1)°	*T* = 173 K
γ = 112.373 (1)°	Block, colourless
*V* = 652.97 (2) Å^3^	0.45 × 0.36 × 0.32 mm

### Data collection

**Table:**

Bruker SMART APEXII CCD diffractometer	3263 independent reflections
Radiation source: rotating anode	3030 reflections with *I* > 2σ(*I*)
Detector resolution: 10.0 pixels mm^-1^	*R*_int_ = 0.023
φ and ω scans	θ_max_ = 28.4°, θ_min_ = 1.9°
Absorption correction: multi-scan (*SADABS*; Bruker, 2009)	*h* = −10→10
*T*_min_ = 0.690, *T*_max_ = 0.746	*k* = −11→11
12250 measured reflections	*l* = −14→14

### Refinement

**Table:**

Refinement on *F*^2^	Secondary atom site location: difference Fourier map
Least-squares matrix: full	Hydrogen site location: difference Fourier map
*R*[*F*^2^ > 2σ(*F*^2^)] = 0.030	H-atom parameters constrained
*wR*(*F*^2^) = 0.081	*w* = 1/[σ^2^(*F*_o_^2^) + (0.0399*P*)^2^ + 0.2911*P*] where *P* = (*F*_o_^2^ + 2*F*_c_^2^)/3
*S* = 1.05	(Δ/σ)_max_ = 0.001
3263 reflections	Δρ_max_ = 0.34 e Å^−^^3^
183 parameters	Δρ_min_ = −0.26 e Å^−^^3^
0 restraints	Extinction correction: *SHELXL2014* (Sheldrick, 2015), Fc^*^=kFc[1+0.001xFc^2^λ^3^/sin(2θ)]^-1/4^
Primary atom site location: structure-invariant direct methods	Extinction coefficient: 0.042 (3)

### Special details

**Table:**

Experimental. ^1^H NMR (δ p.p.m., CDCl_3_, 400 Hz): 8.21 (d, J = 2.04 Hz, 1H), 7.66-7.71 (m, 1H), 7.50-7.57 (m, 2H), 7.32-7.41 (m, 2H), 7.21-7.26 (m, 1H), 3.15 (s, 3H).
Geometry. All esds (except the esd in the dihedral angle between two l.s. planes) are estimated using the full covariance matrix. The cell esds are taken into account individually in the estimation of esds in distances, angles and torsion angles; correlations between esds in cell parameters are only used when they are defined by crystal symmetry. An approximate (isotropic) treatment of cell esds is used for estimating esds involving l.s. planes.

### Fractional atomic coordinates and isotropic or equivalent isotropic displacement parameters (Å^2^)

**Table:**

	*x*	*y*	*z*	*U*_iso_*/*U*_eq_	
Cl1	0.80773 (5)	0.60679 (5)	0.52105 (3)	0.03134 (11)	
S1	0.20658 (4)	0.19225 (4)	0.10339 (3)	0.02090 (10)	
F1	0.22939 (12)	−0.06448 (11)	−0.07604 (8)	0.0324 (2)	
O1	0.70194 (12)	0.36314 (12)	−0.00857 (8)	0.02229 (19)	
O2	0.18502 (13)	0.32691 (13)	0.18956 (10)	0.0283 (2)	
C1	0.44206 (16)	0.27158 (16)	0.08330 (11)	0.0198 (2)	
C2	0.58981 (17)	0.37914 (16)	0.17693 (11)	0.0198 (2)	
C3	0.60633 (18)	0.43369 (17)	0.30452 (12)	0.0220 (3)	
H3	0.5032	0.4009	0.3498	0.026*	
C4	0.77958 (18)	0.53749 (17)	0.36158 (12)	0.0231 (3)	
C5	0.93409 (18)	0.58960 (18)	0.29751 (13)	0.0258 (3)	
H5	1.0502	0.6620	0.3411	0.031*	
C6	0.91896 (18)	0.53654 (18)	0.17133 (13)	0.0255 (3)	
H6	1.0220	0.5701	0.1260	0.031*	
C7	0.74560 (17)	0.43207 (16)	0.11491 (12)	0.0208 (2)	
C8	0.51654 (17)	0.26585 (16)	−0.02553 (12)	0.0201 (2)	
C9	0.44641 (17)	0.17903 (16)	−0.15243 (12)	0.0210 (2)	
C10	0.30426 (18)	0.01690 (17)	−0.17542 (13)	0.0242 (3)	
C11	0.2375 (2)	−0.06907 (19)	−0.29337 (14)	0.0304 (3)	
H11	0.1390	−0.1796	−0.3049	0.036*	
C12	0.3186 (2)	0.0106 (2)	−0.39495 (14)	0.0341 (3)	
H12	0.2744	−0.0450	−0.4778	0.041*	
C13	0.4640 (2)	0.1712 (2)	−0.37641 (13)	0.0311 (3)	
H13	0.5198	0.2238	−0.4466	0.037*	
C14	0.52834 (19)	0.25528 (17)	−0.25675 (12)	0.0244 (3)	
H14	0.6282	0.3649	−0.2451	0.029*	
C15	0.2029 (2)	0.02115 (19)	0.20017 (15)	0.0323 (3)	
H15A	0.2950	0.0697	0.2741	0.048*	
H15B	0.2302	−0.0665	0.1516	0.048*	
H15C	0.0814	−0.0330	0.2276	0.048*	

### Atomic displacement parameters (Å^2^)

**Table:**

	*U*^11^	*U*^22^	*U*^33^	*U*^12^	*U*^13^	*U*^23^
Cl1	0.02955 (19)	0.0403 (2)	0.02092 (16)	0.01176 (15)	−0.00124 (12)	−0.00425 (13)
S1	0.01618 (16)	0.02104 (16)	0.02386 (16)	0.00518 (12)	0.00408 (11)	0.00099 (11)
F1	0.0333 (5)	0.0245 (4)	0.0318 (4)	0.0018 (3)	0.0101 (4)	0.0004 (3)
O1	0.0179 (4)	0.0253 (4)	0.0208 (4)	0.0049 (4)	0.0045 (3)	0.0002 (3)
O2	0.0243 (5)	0.0265 (5)	0.0342 (5)	0.0092 (4)	0.0093 (4)	−0.0024 (4)
C1	0.0168 (6)	0.0198 (6)	0.0214 (6)	0.0054 (4)	0.0033 (4)	0.0016 (4)
C2	0.0176 (6)	0.0194 (6)	0.0223 (6)	0.0068 (4)	0.0031 (4)	0.0021 (4)
C3	0.0211 (6)	0.0247 (6)	0.0212 (6)	0.0095 (5)	0.0042 (5)	0.0024 (5)
C4	0.0245 (6)	0.0250 (6)	0.0199 (6)	0.0102 (5)	0.0007 (5)	0.0000 (5)
C5	0.0191 (6)	0.0268 (6)	0.0270 (6)	0.0054 (5)	−0.0012 (5)	−0.0009 (5)
C6	0.0185 (6)	0.0280 (7)	0.0274 (6)	0.0056 (5)	0.0054 (5)	0.0017 (5)
C7	0.0200 (6)	0.0216 (6)	0.0201 (5)	0.0069 (5)	0.0041 (4)	0.0014 (4)
C8	0.0174 (6)	0.0188 (5)	0.0232 (6)	0.0057 (4)	0.0032 (4)	0.0022 (4)
C9	0.0213 (6)	0.0221 (6)	0.0213 (6)	0.0104 (5)	0.0028 (5)	−0.0004 (4)
C10	0.0230 (6)	0.0235 (6)	0.0265 (6)	0.0092 (5)	0.0051 (5)	0.0009 (5)
C11	0.0286 (7)	0.0263 (7)	0.0319 (7)	0.0083 (6)	−0.0015 (6)	−0.0066 (5)
C12	0.0409 (9)	0.0368 (8)	0.0247 (7)	0.0177 (7)	−0.0020 (6)	−0.0067 (6)
C13	0.0395 (8)	0.0347 (7)	0.0221 (6)	0.0176 (6)	0.0047 (6)	0.0025 (5)
C14	0.0259 (6)	0.0244 (6)	0.0240 (6)	0.0106 (5)	0.0048 (5)	0.0027 (5)
C15	0.0310 (7)	0.0285 (7)	0.0386 (8)	0.0097 (6)	0.0134 (6)	0.0126 (6)

### Geometric parameters (Å, º)

**Table:**

Cl1—C4	1.7425 (13)	C6—C7	1.3800 (18)
S1—O2	1.4913 (10)	C6—H6	0.9500
S1—C1	1.7739 (12)	C8—C9	1.4618 (17)
S1—C15	1.7983 (14)	C9—C10	1.3867 (18)
F1—C10	1.3542 (15)	C9—C14	1.4043 (18)
O1—C7	1.3741 (15)	C10—C11	1.3740 (19)
O1—C8	1.3762 (15)	C11—C12	1.386 (2)
C1—C8	1.3647 (17)	C11—H11	0.9500
C1—C2	1.4420 (17)	C12—C13	1.388 (2)
C2—C7	1.3945 (17)	C12—H12	0.9500
C2—C3	1.3976 (17)	C13—C14	1.3830 (19)
C3—C4	1.3802 (18)	C13—H13	0.9500
C3—H3	0.9500	C14—H14	0.9500
C4—C5	1.3990 (19)	C15—H15A	0.9800
C5—C6	1.3815 (19)	C15—H15B	0.9800
C5—H5	0.9500	C15—H15C	0.9800
			
O2—S1—C1	105.84 (6)	C1—C8—C9	135.10 (12)
O2—S1—C15	105.10 (7)	O1—C8—C9	114.01 (10)
C1—S1—C15	97.80 (6)	C10—C9—C14	117.07 (12)
C7—O1—C8	106.59 (9)	C10—C9—C8	122.54 (12)
C8—C1—C2	106.80 (11)	C14—C9—C8	120.32 (12)
C8—C1—S1	127.53 (10)	F1—C10—C11	117.65 (12)
C2—C1—S1	125.04 (9)	F1—C10—C9	118.49 (12)
C7—C2—C3	119.16 (11)	C11—C10—C9	123.83 (13)
C7—C2—C1	105.31 (11)	C10—C11—C12	117.90 (14)
C3—C2—C1	135.52 (12)	C10—C11—H11	121.1
C4—C3—C2	116.81 (12)	C12—C11—H11	121.0
C4—C3—H3	121.6	C11—C12—C13	120.39 (13)
C2—C3—H3	121.6	C11—C12—H12	119.8
C3—C4—C5	123.12 (12)	C13—C12—H12	119.8
C3—C4—Cl1	118.55 (10)	C14—C13—C12	120.60 (13)
C5—C4—Cl1	118.33 (10)	C14—C13—H13	119.7
C6—C5—C4	120.45 (12)	C12—C13—H13	119.7
C6—C5—H5	119.8	C13—C14—C9	120.18 (13)
C4—C5—H5	119.8	C13—C14—H14	119.9
C7—C6—C5	116.19 (12)	C9—C14—H14	119.9
C7—C6—H6	121.9	S1—C15—H15A	109.5
C5—C6—H6	121.9	S1—C15—H15B	109.5
O1—C7—C6	125.31 (11)	H15A—C15—H15B	109.5
O1—C7—C2	110.42 (11)	S1—C15—H15C	109.5
C6—C7—C2	124.26 (12)	H15A—C15—H15C	109.5
C1—C8—O1	110.87 (11)	H15B—C15—H15C	109.5
			
O2—S1—C1—C8	−138.17 (12)	C1—C2—C7—C6	−179.34 (12)
C15—S1—C1—C8	113.63 (13)	C2—C1—C8—O1	−0.33 (14)
O2—S1—C1—C2	31.48 (12)	S1—C1—C8—O1	170.83 (9)
C15—S1—C1—C2	−76.71 (12)	C2—C1—C8—C9	178.19 (13)
C8—C1—C2—C7	0.43 (14)	S1—C1—C8—C9	−10.6 (2)
S1—C1—C2—C7	−171.01 (9)	C7—O1—C8—C1	0.10 (14)
C8—C1—C2—C3	−178.84 (14)	C7—O1—C8—C9	−178.76 (10)
S1—C1—C2—C3	9.7 (2)	C1—C8—C9—C10	−33.0 (2)
C7—C2—C3—C4	−0.38 (18)	O1—C8—C9—C10	145.51 (12)
C1—C2—C3—C4	178.82 (13)	C1—C8—C9—C14	150.06 (15)
C2—C3—C4—C5	0.61 (19)	O1—C8—C9—C14	−31.45 (17)
C2—C3—C4—Cl1	−179.26 (9)	C14—C9—C10—F1	176.27 (11)
C3—C4—C5—C6	−0.5 (2)	C8—C9—C10—F1	−0.78 (19)
Cl1—C4—C5—C6	179.35 (11)	C14—C9—C10—C11	−1.8 (2)
C4—C5—C6—C7	0.2 (2)	C8—C9—C10—C11	−178.88 (13)
C8—O1—C7—C6	179.14 (12)	F1—C10—C11—C12	−177.47 (13)
C8—O1—C7—C2	0.19 (14)	C9—C10—C11—C12	0.6 (2)
C5—C6—C7—O1	−178.76 (12)	C10—C11—C12—C13	0.8 (2)
C5—C6—C7—C2	0.0 (2)	C11—C12—C13—C14	−1.0 (2)
C3—C2—C7—O1	179.03 (11)	C12—C13—C14—C9	−0.2 (2)
C1—C2—C7—O1	−0.39 (14)	C10—C9—C14—C13	1.59 (19)
C3—C2—C7—C6	0.1 (2)	C8—C9—C14—C13	178.71 (12)

### Hydrogen-bond geometry (Å, º)

**Table:**

*D*—H···*A*	*D*—H	H···*A*	*D*···*A*	*D*—H···*A*
C6—H6···O1^i^	0.95	2.53	3.4756 (16)	176
C14—H14···O2^ii^	0.95	2.44	3.3591 (17)	163

Symmetry codes: (i) −*x*+2, −*y*+1, −*z*; (ii) −*x*+1, −*y*+1, −*z*.
