# Supplementary figures and images for: Crystal structure of 5-chloro-2-(2-fluoro­phen­yl)-3-methyl­sulfinyl-1-benzo­furan
Source: Acta Crystallogr E Crystallogr Commun. 2015 Jul 31;71(Pt 8):o621–2. doi: 10.1107/S2056989015013948 (PMC4571429; doi:10.1107/S2056989015013948)

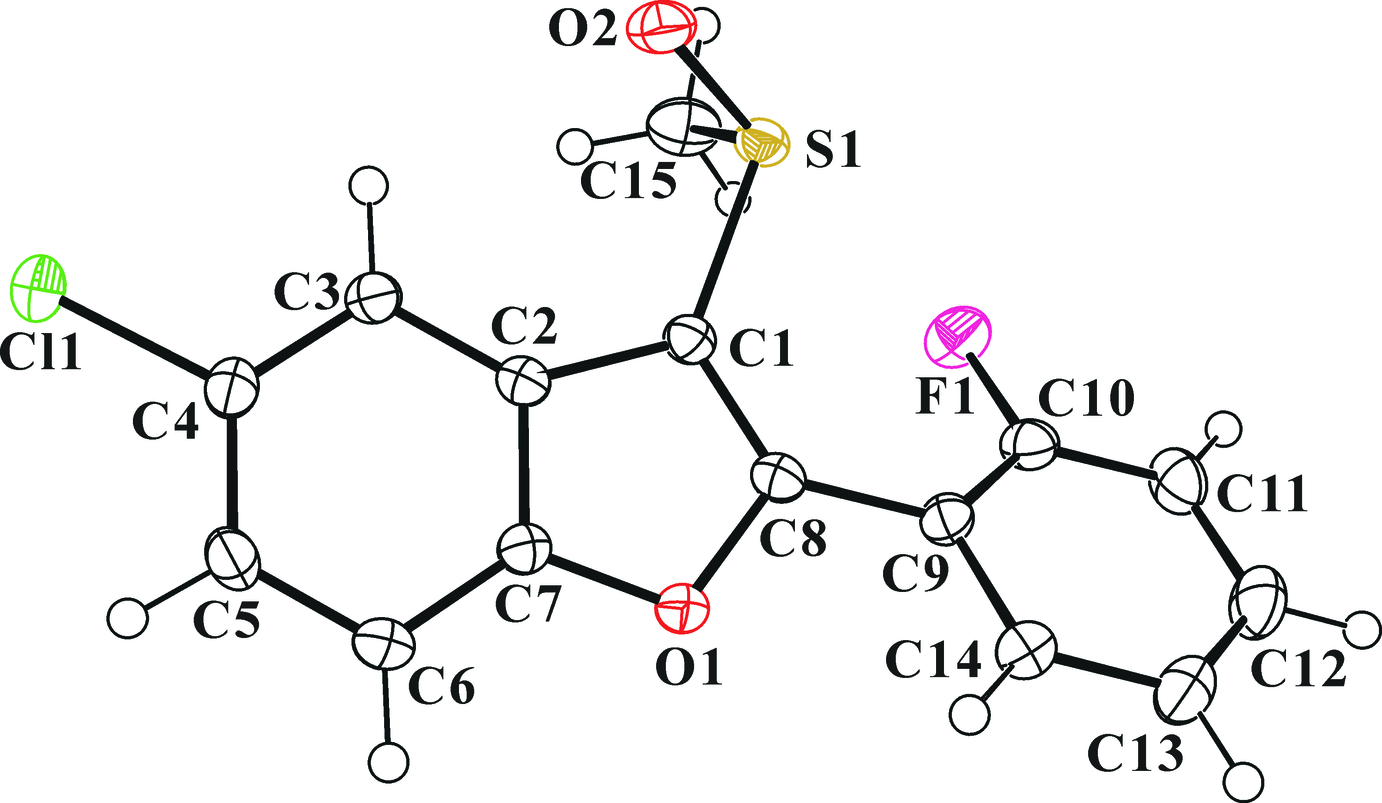

Supplement: Supplementary file 4 [file e-71-0o621-fig1.tif]

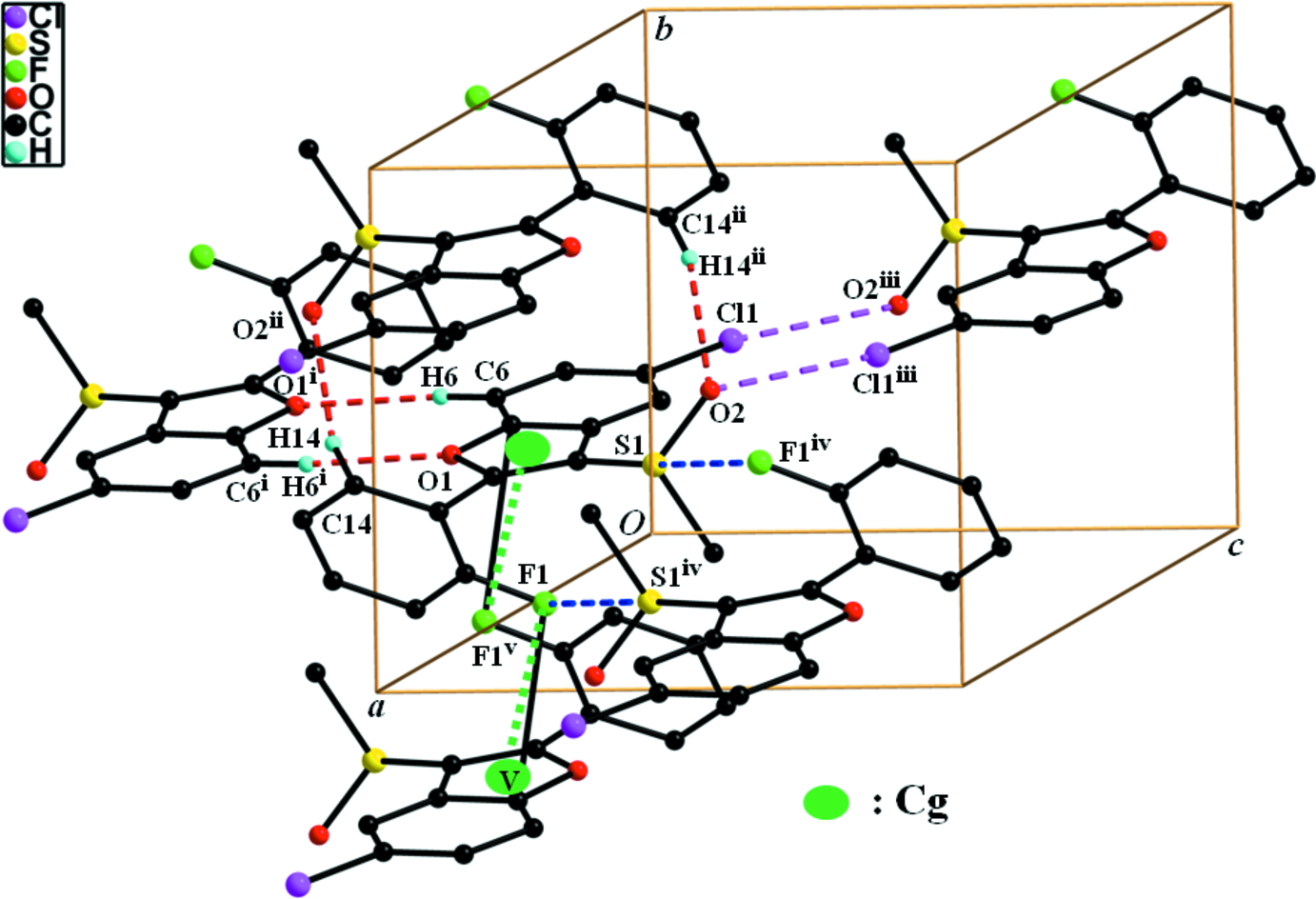

Supplement: Supplementary file 5 [file e-71-0o621-fig2.tif]
